# Marine Macrolides to Tackle Antimicrobial Resistance of *Mycobacterium tuberculosis*

**DOI:** 10.3390/md20110691

**Published:** 2022-11-01

**Authors:** Sukamto S. Mamada, Firzan Nainu, Ayu Masyita, Andri Frediansyah, Rifka Nurul Utami, Mirnawati Salampe, Talha Bin Emran, Clara Mariana Gonçalves Lima, Hitesh Chopra, Jesus Simal-Gandara

**Affiliations:** 1 Department of Pharmacy, Faculty of Pharmacy, Hasanuddin University, Makassar 90245, Indonesia; 2 Department of Pharmaceutical Science and Technology, Faculty of Pharmacy, Hasanuddin University, Makassar 90245, Indonesia; 3 Research Center for Vaccine and Drugs, Research Organization for Health, National Research and Innovation Agency (BRIN), Tangerang Selatan 15318, Indonesia; 4 Research Center for Food Technology and Processing, National Research and Innovation Agency (BRIN), Yogyakarta 55861, Indonesia; 5 Institute of Pharmaceutical Science, King’s College London, London SE1 9NH, UK; 6 Sekolah Tinggi Ilmu Farmasi Makassar, Makassar 90242, Indonesia; 7 Department of Pharmacy, BGC Trust University Bangladesh, Chittagong 4381, Bangladesh; 8 Department of Pharmacy, Faculty of Allied Health Sciences, Daffodil International University, Dhaka 1207, Bangladesh; 9 Department of Food Science, Federal University of Lavras, Lavras 37200-900, Brazil; 10 Chitkara College of Pharmacy, Chitkara University, Punjab, India; 11 Nutrition and Bromatology Group, Department of Analytical and Food Chemistry, Faculty of Food Science and Technology, University of Vigo, Ourense Campus, E32004 Ourense, Spain

**Keywords:** antimicrobial resistance, *Mycobacterium tuberculosis*, structure–activity relationship, marine macrolides, tuberculosis

## Abstract

Tuberculosis has become a major health problem globally. This is worsened by the emergence of resistant strains of *Mycobacterium tuberculosis* showing ability to evade the effectiveness of the current antimycobacterial therapies. Therefore, the efforts carried out to explore new entities from many sources, including marine, are critical. This review summarizes several marine-derived macrolides that show promising activity against *M. tuberculosis*. We also provide information regarding the biosynthetic processes of marine macrolides, including the challenges that are usually experienced in this process. As most of the studies reporting the antimycobacterial activities of the listed marine macrolides are based on in vitro studies, the future direction should consider expanding the trials to in vivo and clinical trials. In addition, in silico studies should also be explored for a quick screening on marine macrolides with potent activities against mycobacterial infection. To sum up, macrolides derived from marine organisms might become therapeutical options for tackling antimycobacterial resistance of *M. tuberculosis*.

## 1. Introduction

Tuberculosis (TB) has become a global burden for years, especially in the developing areas of the world. A recent report by the World Health Organization showed that a total of 1.5 million TB-linked deaths was recorded in 2020 [1]. From the same report, it was also reported that the efforts for treating patients suffering from TB experienced an unprecedented obstacle because of COVID-19. This pandemic has limited access to provide the appropriate treatments for TB patients. For example, the number of patients treated with a drug regimen for drug-resistant TB dropped by 15%. Furthermore, the number of those receiving TB-preventive treatments also experienced a decrease of 21% [1]. 

Like other microbial infections, treatment of TB is always overshadowed by the case of resistance. It has been demonstrated that *Mycobacterium tuberculosis*, the causative agent of TB, has developed a number of modes used to evade the therapeutical actions of the current TB drugs [2,3,4]. Consequently, new strategies should be applied to counter this issue.

One of the reasonable strategies is to find and develop new TB drugs. It is noteworthy that the new TB drugs should try to explore drug candidates with a different core structure, potency, or mechanism than the currently used TB drugs. At this point, the efforts for discovering new TB drugs should examine the potencies of drug candidates originating from marine sources. However, this strategy is limited by the fear of excessive exploitation of natural marine resources. To counter this obstacle, chemical synthesis equipped with the knowledge of the structure–activity relationship could provide a breakthrough to avoid unexpected damages to the marine environment.

Macrolides are a type of polyketide antibiotic. They obstruct protein synthesis by binding to the bacterial 50S ribosomal subunit at the peptidyl transferase center formed by 23S rRNA [5]. Since the discovery of the first macrolide, pikromycin, in 1950 from an actinomycete, many macrolides have been discovered and classified into different groups [6]. Each macrolide molecule is distinguished by three structural elements: a macrocyclic lactone ring, multiple ketone and hydroxyl groups, and two deoxy sugars linked by a glycosidic bond [7]. The number of carbons in the lactone ring divides macrolides into several groups, including 12-membered rings (i.e., methymicin, neomethymycin, and litorin), 14-membered rings (i.e., erythromycin A and F, oleandomycin, anthracimycin, clarithromycin, roxithromycoin, and sporamicin), 15-membered rings (i.e., azithromycin), and 16-membered rings (i.e., tylosin, josamycin, kitasamycin, spiramycin, and midecamycin).

Although their use in the treatment of TB is not as frequent as the other TB drugs, e.g., isoniazid, ethambutol, and pyrazinamide, macrolides might act as a therapeutical option in a TB regimen. Several macrolides, e.g., erythromycin, azithromycin, and clarithromycin, have been used in treating a number of microbial infections, including TB [8]. However, it has been reported that the effectiveness of these macrolides in fighting the microbes decreases over time [9,10,11]. Therefore, the seeking of new macrolides from various sources, including from the ocean, with a promising antimicrobial action is pivotal. 

As reviewed by Das et al., marine macrolides possess various potential biological activities, such as anti-inflammatory and anticancer activities, including their potent ability as antimicrobial agents [12]. Here, we review several macrolides obtained from marine organisms showing a promising potency as antimycobacterial agents. Although several studies have reported the presence and identification of marine-based macrolides, to the best of our knowledge, no studies have tried to collect valuable information regarding the specific uses of marine macrolides as antimycobacterial agents. At this point, this review aims to fill that gap and provides the lost brick needed to comprehensively understand the antimycobacterial potencies of marine-based macrolides. 

## 2. Materials and Methods

Available articles from three bibliographical databases, e.g., Google Scholar, Scopus, and PubMed, were searched. The search criteria were represented by the keywords used, i.e., (“macrolide” OR “macrolides”) AND (“marine” OR “sea” OR “ocean” OR “coral” OR “algae” OR “sponge”) AND (“antimycobacterial” OR “mycobacterial” OR “*Mycobacterium*” OR “anti-*Mycobacterium tuberculosis*” OR “*Mycobacterium tuberculosis*” OR “*M. tuberculosis*” OR “tuberculosis” OR “mycobacterial infections”).

## 3. Structure–Activity Relationship of Macrolides

Structure–activity relationship (SAR) studies explore the relationships between the chemical structure and biological activity of a molecule [13]. Various SAR studies of macrolides have been reported to identify the correlation of functional elements essential for maintaining antibacterial activity [14,15]. Structurally, macrolides contain a macrocyclic lactone of different ring sizes and are decorated with one or more deoxy-sugar or amino sugar residues [16]. Macrolides display broad-spectrum activity against many Gram-positive bacteria by binding to bacterial 50S ribosomal subunits and interfering with the synthesis of protein [17]. The first-generation prototypical macrolide, erythromycin, is a naturally occurring antibiotic produced by *Streptomyces erythreus*, currently reclassified as *Saccharopolyspora erythraea*. Erythromycin has several co-metabolites (A, B, C, D), of which the derivative A (erythromycin A) is the most dominant and most used product in therapy [14,18].

**Figure 1:**
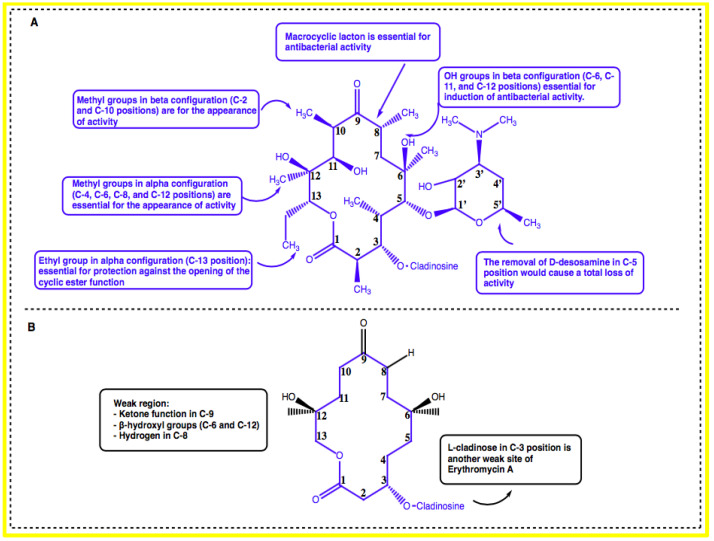
(**A**) Structure–activity relationship study of erythromycin. (**B**) The weak regions of erythromycin.

Erythromycin possesses a 14-membered macrolactone ring called erythronolide and is attached with L-cladinose (a neutral sugar in C-3 position) and D-desosamine (an amino sugar in C-5 position). The integrity of erythronolide including 9-oxime is essential for antibacterial activity [14]. Moreover, the presence of the methyl groups in an alpha configuration in the C-4, C-6, C-8, and C-12 positions as well as the methyl groups in a beta configuration in the C-2 and C-10 positions is also essential for the appearance of activity. In addition, the hydroxyl groups in beta configuration in the C-6, C-11, and C-12 positions are essential for induction of antibacterial activity. Furthermore, the removal of D-desosamine in the C-5 position would cause a total loss of activity. The integrity of aglycone could be maintained by the presence of an ethyl group in an alpha configuration in the C-13 position, which would be essential for protection against the opening of the cyclic ester function (Figure 1A) [14,19]. 

The weak regions (inactivation sites) of erythromycin A have also been identified by SAR studies. The ketone function in the C-9 position, beta-hydroxyl groups in the C-6 and C-12 positions, and hydrogen in the C-8 position are responsible for the formation of inactive hemiketal and spiroketal derivatives, which are responsible for the digestive intolerance of erythromycin A. Additionally, the presence of L-cladinose in the C-3 position is another weak site of erythromycin A (Figure 1B). This entity could be hydrolyzed in gastric-acid medium to form an inactive 3-hydroxy erythromycin A derivative, which is responsible for the appearance of drug-resistant germs [14]. 

The second-generation macrolides, clarithromycin, roxithromycin, and dirithromycin, are semisynthetic derivatives of the 14-membered ring-macrolide erythromycin A and the 15-membered one (azithromycin). Clarithromycin has a methoxy group at the C-6 position of the lactone ring. Flurithromycin has a fluorine atom at the C-8 position (alpha to the ketone carbonyl group) and roxithromycin has an etheroxime chain at the C-9 position. Azithromycin has an azalide group at the C-9a position. These derivatives contain all modifications at the C-6 or C-9 positions of the macrocyclic lactone, consequently preventing the formation of hemiketal and spiroketal derivatives [20].

Existing 14-, 15-, and 16-membered macrolides, although effective for other bacterial infections, did not display significant efficacy in treating tuberculosis [20]. Studies of the activity of macrolides against *Mycobacterium tuberculosis* are very restricted. Recently, Zhang et al. studied the antituberculosis potency of clarithromycin. They reported that a single methyl group in clarithromycin improves its potency against *M. tuberculosis*. Interestingly, they also found that the allosteric dynamics of A2062 by ribosome–clarithromycin complex may have great potential to increase the drug efficacy and may help to design the next generation of antituberculosis drugs to fight against multidrug-resistance tuberculosis [21]. 

**Figure 2:**
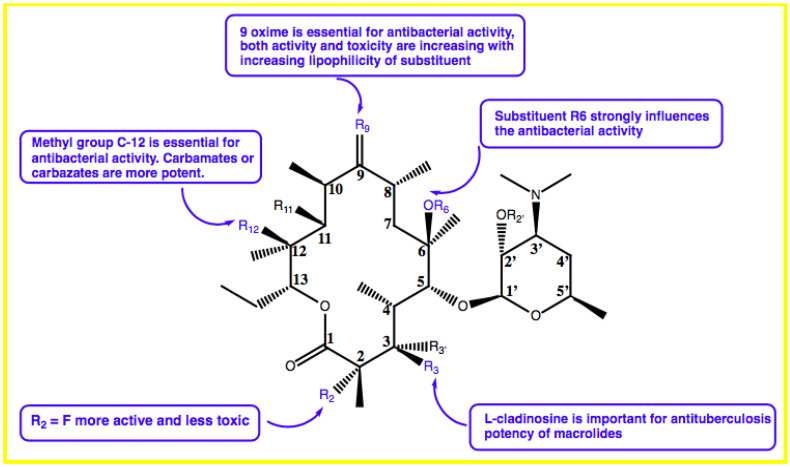
Structure–activity relationship study of macrolides.

Zhu et al. found that the appropriate substitutions on the C-9, C-11, C-12, or C6 positions in the macrolactone ring have good activity against *M. tuberculosis*. This group also showed that L-cladinosine located in the C-3 position is critical for macrolides in exerting their anti-mycobacterial activities (Figure 2) [20]. 

## 4. Biosynthesis of Marine Macrolides

### 4.1. Biosynthesis Challenges

Macrolides are extremely important as pharmaceutical leads, yet the significant slowing of innovative chemical development has become a major concern. Theoretically, macrocyclic systems can be created by cyclizing open, long-chain progenitors or cleaving internal polycyclic bonds. However, due to the structural complexity of macrocyclic natural compounds, difficulties in derivatizing them by chemical synthesis pose a barrier to pharmaceutical development. Moreover, several problems exist during the synthesis process to achieve the desired compounds. Thus, genetic modification can generate a huge number of congeners from known valuable natural substances.

Generally, the biosynthesis of marine macrolides, typified by erythromycin derivatives, is catalyzed by modular type I polyketide synthase (PKS) and modified by tailoring enzymes such as cytochrome P450 (CYP450), glycosyltransferase (GT), and oxidation enzymes such as monooxygenase (MO), methyltransferase (MT), and oxidoreductase (OR) [22,23,24]. The type I PKSs are multi-modular enzymes with non-iterative catalysis of one cycle of polyketide-chain elongation that are liable for consecutive condensation of activated coenzyme A (CoA) thioesters, including acetyl-CoA, propionyl-CoA, malonyl-CoA, or methylmalonyl-CoA. Each module minimally contains a set of functional domains, acyltransferase (AT), β-ketosynthase (KS), and acyl carrier protein (ACP) that are required for growing a polyketide chain and generating a β-ketoacyl-S-ACP intermediate [25,26]. In addition, the modules may also contain domains that consecutively modify the β-keto group to a hydroxyl group (ketoreductase, KR), a double bound (dehydrase, DH), and a single bond (enoyl reductase, ER) [26,27]. The ACP provides both intermediates of polyketide and building blocks to the catalytic domains for loading, extension, and processing of the keto-group utilizing thioester (TE) linkages and a tethered phosphopantetheine arm [28]. Mostly, a TE domain in the terminal module releases the intermediates, which are fully processed via cyclization or hydrolysis of macrolactone [26,29]. 

**Figure 3:**
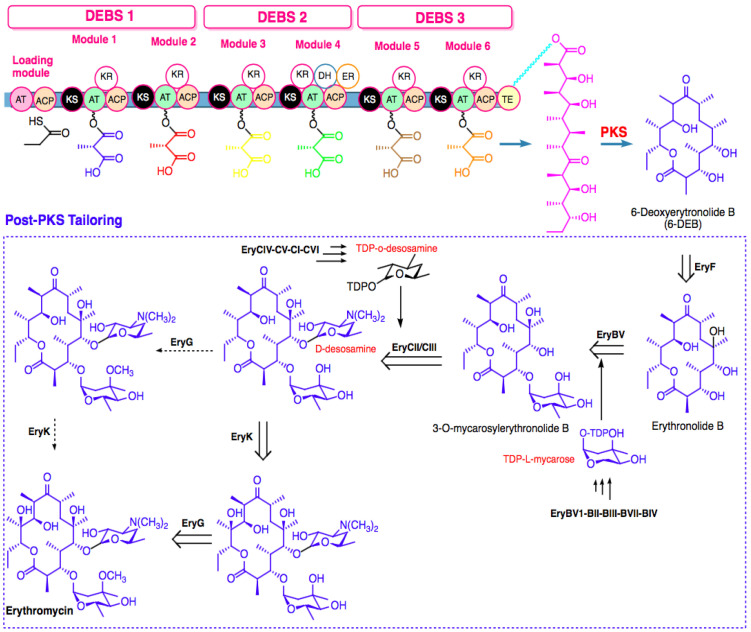
Biosynthesis-scheme assembly of erythromycin.

After PKS-mediated biosynthesis of aglycones, the post-PKS modification extensively occurs to generate the structure of the final macrolide. Figure 3 shows the precursor-directed biosynthesis of erythromycin, which is divided into two stages [26]. First, 6-deoxyerythronolide PKS (DEBS), the modular PKS complex, catalyzes the sequential condensation of six methylmalonyl-CoA precursors and one propionyl-CoA precursor to generate macrolide 6-deoxyerythronolide B (6-dEB). Second, 6-dEB is converted to erythronolide B by EryF hydroxylase. Then, EryBV glycosyltransferase transfers L-mycarose to erythronolide B, forming 3-O-mycarosylerythronolide B. EryCII activates EryCIII, completing the attachment of two deoxy sugars to the aglycone ring by transferring D-desosamine to C-5 hydroxyl, yielding erythromycin D as the first bioactive intermediate. Subsequently, methylation of 3′′-OH of L-mycarose by EryG methyltransferase and hydroxylation of C-12 by EryK hydroxylase generates the final product (erythromycin) [26].

Fundamentally, there are four steps throughout biosynthesis for diversification of a macrolide: (1) choice of the length of the chain and building block; (2) reduction and stereochemical arrangement of β-keto intermediates, including primary cyclization, branching, and alkylation; (3) rearrangement and secondary cyclization; and (4) tailoring of post-PKS [22,30]. Furthermore, it has been reported that the AT domain of modular PKSs controls the specific extender unit selected by each module, which naturally offer large portions of the polyketide structure, as these extender units are gathered into scaffolds of natural product. The ATs were able to discriminate between extender units to the PKS in the producing organism [31].

Biosynthesis of macrolides utilizing modular type I PKSs are usually bounded in scope and utility due to the limited substrate specificity of the polyketide biosynthetic machinery [26]. Recent information on the protein structure of enzymes and the advanced technique of genetic manipulation presents an immense opportunity for fine-tuning the step of post-PKS to yield structurally diversified or novel macrolides [32]. The diverse modularity in the genetic architecture of PKSs presents sufficient reason for expecting feasibility for engineering the enzymes to obtain new drug candidates by combinatorial biosynthesis [22,33]. 

Shinde et al. evaluated the combinatorial biosynthesis of glycosylated derivatives of a 12-membered macrolide by utilizing *Streptomyces venezuelae* YJ003 [34]. *S. venezuelae* has been developed as an important host for the combinatorial biosynthesis of new macrolides due to its amenability to genetic manipulation and faster growth rate than other streptomycetes. Their results revealed that combinatorial biosynthesis has promising potential to generate new glycosylated macrolides with improved antibacterial activities. L-rhamnosyl-10-deoxymethynolide exhibited outstanding activity against clinically isolated erythromycin-resistant pathogenic strains, as well as erythromycin-susceptible strains relative to YC-17 and its other analogs [34]. Similarly, by utilizing *S. venezuelae*-based combinatorial biosynthesis machinery, Jung et al. successfully revealed the bioconversion of 12-, 14-, and 16-membered ring macrolactones, including 10-deoxymethynolide, narbonolide, and tylactone, respectively, to glycosylated macrolides. The biosynthesis of TDP-3-dimethyl-D-chalcose or TDP-L-rhamnose together with DesVII/DesVIII, a novel narbomycin derivative (novel analog) decorated with L-rhamnose or 3-O-demethyl-D-chalcose, were obtained. These compounds showed greater antibacterial activity than narbomycin and the clinically relevant erythromycin [35].

Additionally, in a study by Ye et al., three 22-membered macrolides were discovered by deciphering the streamlined genome of mangrove-derived *Streptomyces* sp. HM190, which is a marine actinomycete. They found that PKS genes S1–S8 were proposed to be responsible for the production of three 22-membered macrolides. A total of 30 biosynthetic gene clusters (BGCs), especially gene cluster 5, were responsible for biosynthesis of the macrolide in a strain-specific 126,331bp genomic island belonging to the left-arm region [36]. 

The construction of macrocyclic structures of marine macrolides remains a challenging problem in medicinal chemistry [37]. However, several new synthetic methods have been discovered to overcome this hurdle and mostly emphasize the key macrolide ring-forming reactions [37]. For example, the total synthesis of borrelidin has been achieved by utilizing a samarium-(II) iodide-mediated intramolecular Reformatsky-type reaction for macrocyclization at C11–C12 after esterification between two segments [38]. Additionally, Terwilliger et al. reported the first synthesis of divergolide I (a naphthoquinone ansamycin). They demonstrated that the biomimetic cyclization of a protodivergolide (a macrocyclic precursor) could be surprisingly enantioselective and relatively short (less than 20 linear steps) [39].

### 4.2. Metabolic Engineering

The main constraint for macrolide biosynthesis in the producer-host is affluent availability of cofactors and precursors, which generally derive from primary metabolism, including glycolysis, tricarboxylic acid cycle (TCA), pentose phosphate pathway, and amino-acid/nucleic-acid metabolism [22,40], as well as the lower expression level of biosynthetic genes and regulation of the biosynthetic gene/genes [41]. Basically, correlation and regulation of precursor supply for increasing the number of natural products are focused on metabolism of carbohydrates, intracellular cofactor supplies, and fatty-acid precursors [42]. Numerous studies have been reported to illustrate the genetic circuit-guided pathway engineering approaches for increasing the important secondary metabolites (Table 1), such as heterologous overexpression of the S-adenosyl-L-methionine (SAM) synthetase metK and enhanced production of pikromycin, avermectin, and actinorhodin [43,44]. 

One metabolic-engineering strategy for enhanced production of erythromycin involves blocking the flow of propionate into the TCA cycle through a *mutB* knockout in the industrial *S. erythraea* strain HL3168 E3 [45]. Similarly, the engineering of the methylmalonyl-CoA metabolite node of *S. erythraea* through duplication of the mmCoA mutase operon (meaA, mutB, meaB, mutR) led to an elevated level of erythromycin in the oil-based medium [46]. On the other hand, from comparative proteomic and transcriptomic approaches, a putative regulatory protein (SACE_5599) exhibited significantly higher expression in the industrial *S. erythraea* strain ABE1441 as compared to the wild-type strain and was also involved in erythromycin production [47]. 

Furthermore, metabolic engineering was employed to improve the formation of novel erythromycin analogues by altering the tailoring pathway modularity in the biosynthesis of erythromycin analogs heterologously engineered in *E. coli* [48].

Overall, three steps are critical, including (1) bioinformatics analysis of assembly-line enzymes. The first step toward successful assembly-line enzyme engineering is a thorough understanding of the enzyme's domains and modules. Because of their huge size, these genes are frequently misannotated in public genome databases, and their highly repetitive sequences tend to induce problems in open-reading frame annotation or in next-generation sequencing itself. (2) Heterologous expression vector construction: The expression vector is essential for heterologous expression to be successful. Because of their ease of use in gene manipulation, *E. coli*-*Streptomyces* shuttle vectors are the most commonly used. (3) Modification: Due to the lack of a robust DNA restriction system, the *Streptomyces* strain has been widely employed as a heterologous expression host [49,50].

## 5. Mechanism of Resistance of *Mycobacterium tuberculosis*

**Figure 4:**
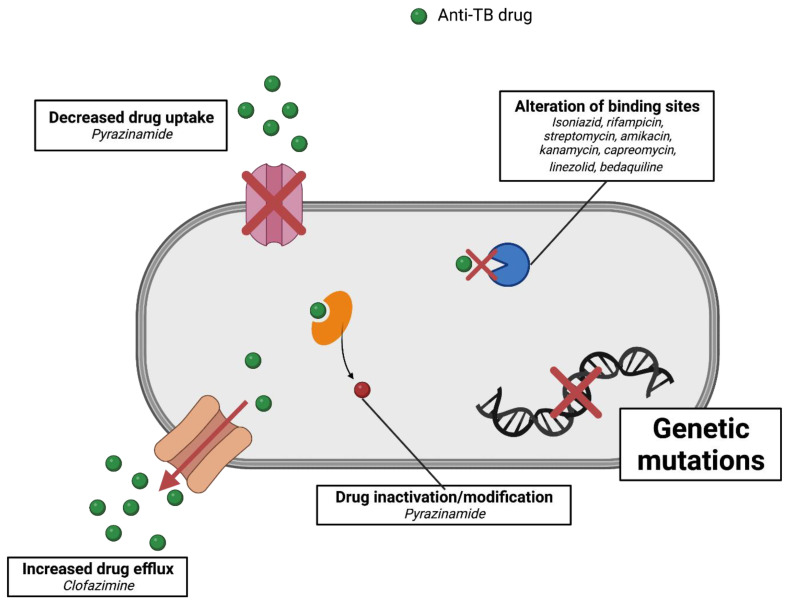
Summary of the mechanisms of resistance of *M. tuberculosis* to particular anti-TB drugs.

Tuberculosis is a world health problem that is exacerbated by the emergence of resistant strains of *M. tuberculosis*. The pattern of resistance found in TB cases can be classified based on the class of drugs involved. Multidrug-resistant TB (MDR-TB) is a case of TB caused by strains that are resistant to two first-line drugs, namely, isoniazid and rifampicin [57]. A more resistant strain was discovered in 2006, termed extensively drug-resistant TB (XDR-TB). In the case of XDR-TB, resistance increases to include fluoroquinolones and at least one of the second-line injectables, including amikacin, capreomycin, and kanamycin. Recently, a more worrying case occurred, namely, the emergence of TB strains that are not sensitive to all available treatments, also known as totally drug-resistant TB (TDR-TB) (Figure 4) [58,59].

A deeper understanding of the mechanism of resistance in *M. tuberculosis* strains is imperative to aid the development of new drugs and the detection of resistance levels in patients. Early detection is very important to ensure better disease management and spread prevention. Principally, *M. tuberculosis* resistance is acquired through mutations in genes that play a role in the expression of drug targets [4]. Patients can become infected with resistant TB through two scenarios. First, the host becomes infected with resistant strains, resulting in primary-drug resistance. Second, resistance develops during disease progression due to the emergence of new mutations, also known as secondary-drug resistance [2,3]. Furthermore, the mechanisms of resistance of *M. tuberculosis* to particular anti-TB drugs are discussed in the following sections and summarized in Figure 4.

### 5.1. Isoniazid

Isoniazid (INH) is one of the first-line drugs in the treatment of TB. This compound inhibits the synthesis of the cell wall of *M. tuberculosis* by preventing the formation of mycolic acid [60]. This process is related to the inhibitory activity of the inhA protein. INH is a prodrug that is activated by the enzyme KatG, which is produced intracellularly by *M. tuberculosis*. This enzymatic process pairs INH with NADH and renders it active. Therefore, mutations in the *inhA, katG*, and *ndh* genes are considered to be the main mechanisms of resistance to INH [61,62]. In particular, mutations in *katG*, which are more common in MDR cases, are associated with high levels of resistance [63]. Meanwhile, mutations that occur in inhA will change the structure of the INH-binding target site [64]. Mutations in *inhA* also affect the performance of other drugs that have a similar therapeutic action, such as ethionamide [65]. Mutations in the *ahpC* promoter gene that plays a role in the synthesis of alkyl hydroperoxidase reductase enzymes were also considered as markers of resistance to INH. However, it was later found that this gene modification compensates for changes in catalase–peroxidase activity and is not directly related to INH resistance [66]. Mutations in *kasA* have also been reported in INH-resistant strains, although their exact role remains unclear [67].

### 5.2. Rifampicin

Rifampicin plays a key role in the treatment of drug-sensitive TB (DS-TB) because of its effectiveness against both active and slow-metabolizing bacilli [68]. Rifampicin acts by binding to the beta subunit of RNA polymerase, inhibiting mRNA elongation [69]. Mutations in the gene encoding the protein, *rpoB*, have been reported as the main mechanism of resistance to rifampicin. The mutation is particularly clustered at codon 507–533, also known as the rifampicin-resistance-determining region. Approximately 96% of cases of rifampicin resistance are associated with this mutation [11,70]. Interestingly, single resistance to rifampicin is rare. Cross-resistance to other drugs, particularly INH, has been reported frequently [71]. Therefore, *rpoB* mutations are often used as a surrogate marker for the determination of MDR strains. This mutation also directly reduces the effectiveness of other rifamycin-derived drugs [9].

### 5.3. Ethambutol

Ethambutol inhibits arabinogalactan synthesis in the cell walls of actively multiplying bacilli [72]. Resistance to ethambutol is related to disruption of the *embB* gene encoding the enzyme arabynosyl transferase, specifically at codon 306 [73]. However, about one-third of ethambutol-resistant isolates were found to have no alterations to *embB306*, suggesting that another possible mechanism is involved [2,4]. Previous reports have shown that simultaneous mutations between *embB/embC* and a gene involved in the biosynthesis of decaprenylphosphoryl-β-D-arabinose lead to variable changes in ethambutol MIC. It is important to note, however, that *embB* mutations alone elicit variable, but not high, resistance levels [10]. Another gene reported to play a role in ethambutol resistance is *ubiA*, which encodes decaprenyl-phosphate 5-phosphoribosyltransferase synthase. This enzyme is also involved in the synthesis of bacterial-cell walls. Mutations in *ubiA,* when co-occurring with embB mutations, can lead to high levels of ethambutol resistance [74].

### 5.4. Pyrazinamide

Pyrazinamide is part of the first-line regimen in the treatment of TB. An interesting feature of this drug is its ability to target semi-dormant bacilli in TB lesions [75]. The use of this drug allows a reduction in the duration of TB treatment to 6 months. Pyrazinamide penetrates bacterial cells and is activated by pyrazinamide/nicotinamidase to the active form of pyrazinoic acid [76]. Normally, pyrazinoic acid would be subject to the efflux mechanism. However, under acidic conditions, as in TB lesions, protonated pyrazinoic acid allows re-entry into cells [77]. Resistance to pyrazinamide is generally associated with mutations in the *pncA* gene encoding the enzyme pyrazinamide/nicotinamidease [78]. However, a small percentage of resistant isolates showed no mutations in the *pncA* gene, suggesting that other types of mutations may be involved [79].

### 5.5. Streptomycin

Streptomycin inhibits bacterial protein synthesis by binding irreversibly to the 30s ribosomal subunit and is active in slow-growing bacilli [80]. This antibiotic was the first drug introduced in the treatment of TB and resistance to it is growing rapidly [81]. Streptomycin-resistant strains have been reported to have mutations in the *rpsL* and *rrs* genes, which are associated with ribosomal protein rRNA. Nearly three-quarters of the resistant isolates were found to have this mutation [82].

### 5.6. Fluoroquinolones

Fluoroquinolones are second line in the treatment of TB. This class of antibiotics prevents bacterial-cell replication by inhibiting DNA gyrase. Ciprofloxacin and ofloxacin are examples of previous-generation drugs that have been used for TB [83]. Two fourth-generation fluoroquinolones, moxifloxacin and gatifloxacin, are new therapeutic candidates for DR-TB [84]. *gyrA* and *gyrB* are the genes that code for DNA gyrase; thus, mutations in them could result in resistance to quinolones. Single mutations in *gyrA* or *gyrB* lead to significant resistance, and multiple mutations lead to a higher increase in MIC [85,86].

### 5.7. Second-Line Injectables

Amikacin and kanamycin (aminoglycosides) and capreomycin (cyclic polypeptides) belong to the second-line injectable group for the treatment of TB. These drugs have the same target of action, despite the different antibacterial classes [2,3]. Therefore, the mechanism of resistance to these compounds is also interrelated. These drugs act by inhibiting protein synthesis through the modification 16s rRNA on the bacterial ribosome. Mutations in the *rrs* gene are associated with high levels of resistance. The most common molecular mechanism is the A-G polymorphism at position 1401 of the *rrs* gene [87]. For capreomycin, mutations in the *tylA* gene involved in ribose methylation in rRNA were also found to trigger additional resistance [88]. Cross-resistance among these three drugs has also been reported [89,90].

### 5.8. Para-Amino Aalicylic Acid (PAS)

Previously one of the first choices in the treatment of TB, this para-amino benzoic acid analogue is now part of second-line drugs. The bactericidal activity of the drug is obtained through inhibition of folate synthesis [3]. Approximately 40% of PAS-resistant strains exhibit mutations in the *thyA* gene [91]. Also recently, mutations in the *folC* gene encoding dihydrofolate synthase were found to correlate with resistance to PAS in laboratory isolates of *M. tuberculosis* [92].

### 5.9. Novel and Repurposed Drugs

Several new drugs have been applied in the treatment of TB. However, although relatively new, resistance has been reported with some of these drugs. Linezolid, an oxazolidinone, is an early-stage inhibitor in protein synthesis. The drug binds to the V domain of the 50s subunit of the bacterial ribosome [93]. Two genes found to be associated with linezolid resistance were *rrl* and *rplC* [94]. Another drug that has also been used as a new agent for TB is bedaquiline from the diarylquinolines class. Bedaquiline acts by inhibiting bacterial-cell respiration by targeting ATP synthase in *M. tuberculosis* [95]. Mutations in the *atpE* gene related to this process have been associated with high levels of resistance to bedaquiline [96,97]. 

One example of a repurposed drug in TB therapy is clofazimine, which was previously used in the treatment of leprosy. This drug has now become part of a short-course treatment regimen based on WHO recommendations. The exact mechanism of this drug has not been established. However, previous studies have suggested that the formation of reactive oxygen species after clofazimine is reduced by NADH dehydrogenase [98]. One mechanism associated with resistance to clofazimine is an off-target mutation in *rvo678* that causes increased efflux of the drug pumped out of mycobacterial cells [99].

## 6. Marine Macrolides to Counter *Mycobacterium tuberculosis* Resistance

**Figure 5:**
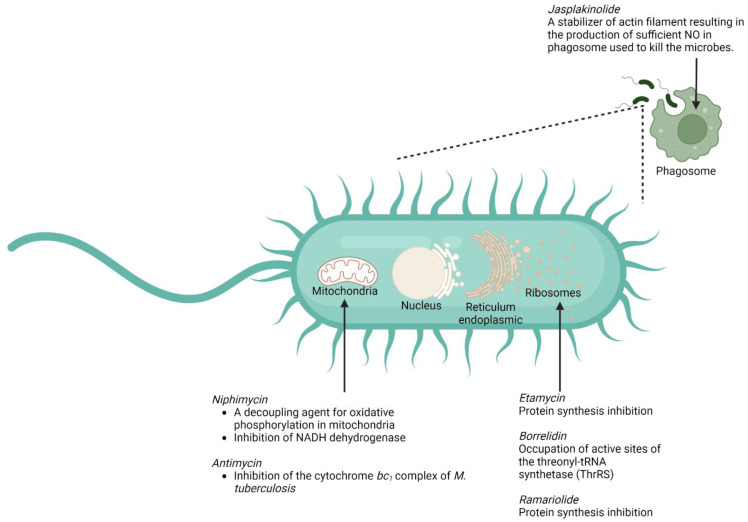
Summary of the mechanisms of action of several marine macrolides against *M. tuberculosis*.

**Figure 6:**
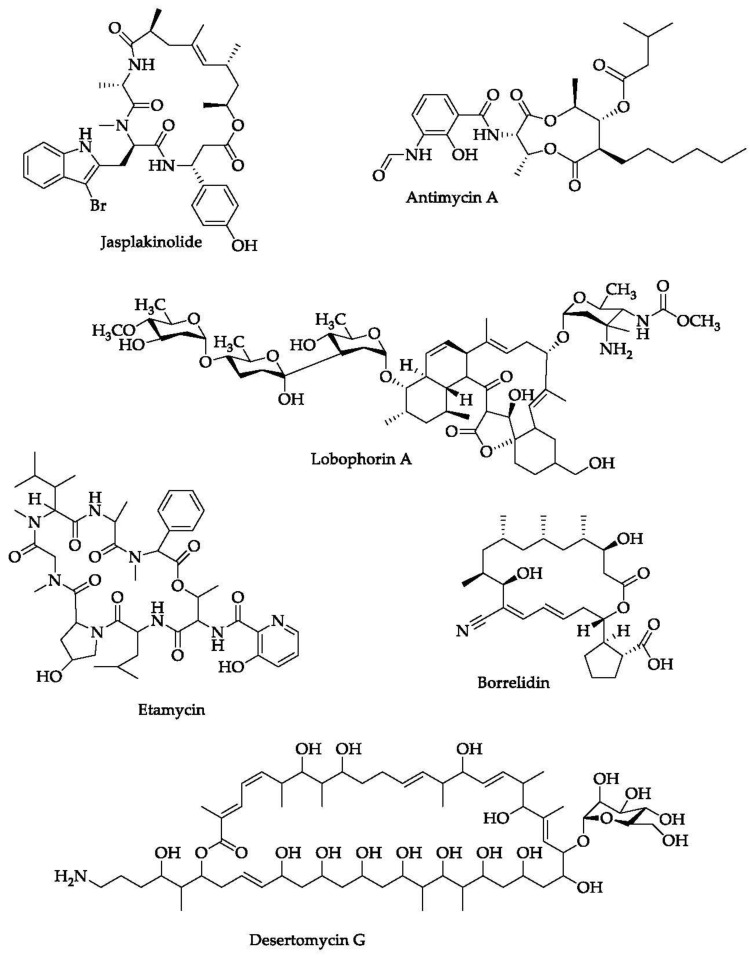

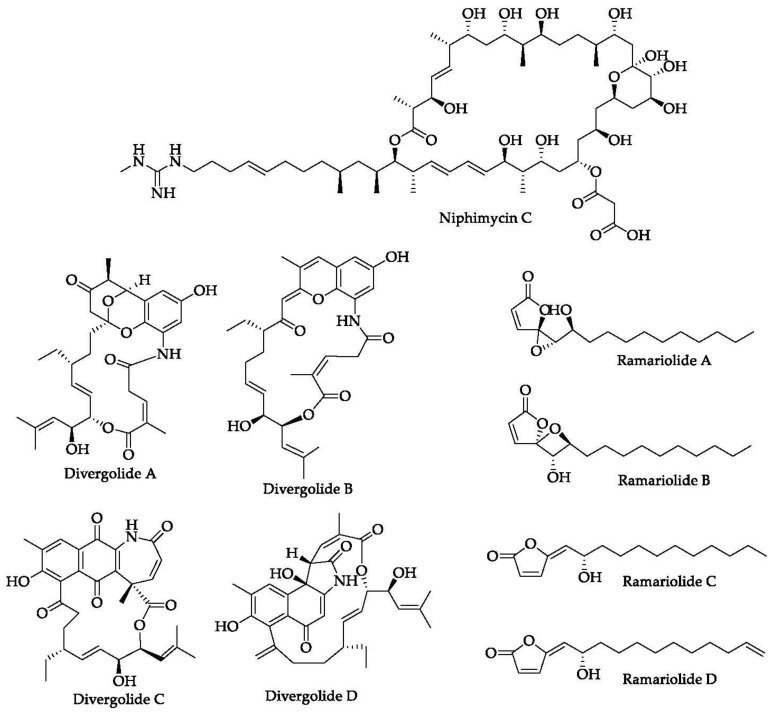
Chemical structures of several macrolides isolated from marine organisms.

In this section, we have listed several compounds classified as macrolides isolated from marine organisms (Figure 5 and Table 2). These macrolides, either in a single administration or in combination with other compounds, have a promising activity for tackling mycobacterial infection so that they can hopefully provide a breakthrough for countering *M. tuberculosis*-resistance cases. The structures of the listed marine macrolides are presented in Figure 6.

Although disturbance of bacterial-protein synthesis in ribosomes is still the main mechanism by which the macrolides (e.g., etamycin, ramariolide, and borrelidin) exert their antimycobacterial action [118,119,120], several marine macrolides also offer a number of relatively different ways to act as an antimicrobe. One of those mechanisms is associated with the ability of the macrolide (jasplakinolide) to disturb the regulation of actin filament, leading to the success of the macrophages in killing the bacterium [101]. Another mechanism that might be exploited by the marine macrolide (antimycin and niphimycin) is linked to their ability to perturb the activity of the mitochondria in *M. tuberculosis* [106,107,121]. The summary of the putative mechanisms by which several marine macrolides exert their antimycobacterial actions is provided in Figure 5.

Most of the compounds listed above show their antimycobacterial activities. However, those activities have been confirmed through in vitro studies using various *M. tuberculosis* variants. Based on the MIC values, niphimycin (4–16 μg/mL) and etamycin (0.097–25 μg/mL) showed more potency in inhibiting the growth of several tested mycobacteria relative to the other compounds. 

Moreover, both entities demonstrated fewer cytotoxic effects compared to the related control groups, indicating their safety in the tested models. For example, the cytotoxic activity of niphimycin C and niphimycin Iα on K562, HepG2, MCF-7, and HeLa cells was 8.5–10.2 μM and 6.8–23.9 μM, respectively, whereas the standard drug (doxorubicin) yielded 1.1–3.5 μM.

As these results were collected through various in vitro models, further in vivo and clinical studies must be conducted to confirm their efficacy and safety. To provide a quick screening on macrolides extracted from marine sources, in silico studies should also be considered. Although the others have less potency in inhibiting the growth of the mycobacteria, these compounds have the potential to be modified structurally to form the related compounds with better efficacy and safety properties.

**Figure 7:**
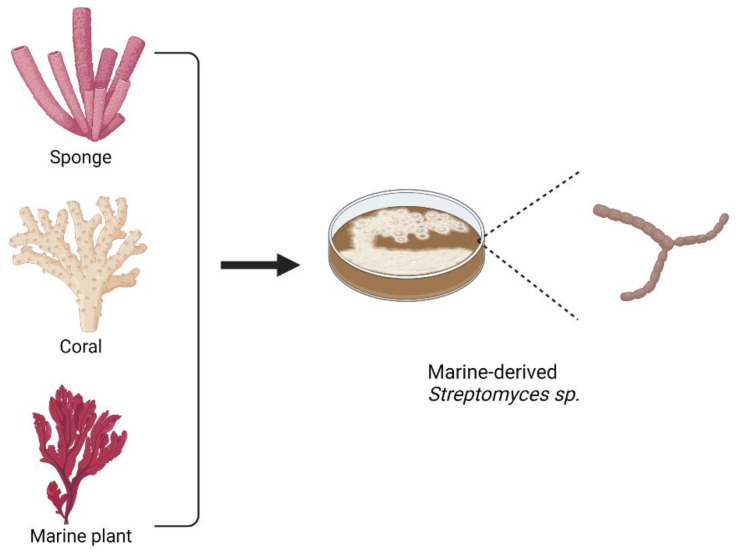
Examples of organisms as the main sources for obtaining macrolide-producing marine microorganisms, including *Streptomyces* sp.

From the list, marine *Streptomyces* sp. becomes the major source for marine macrolides. In addition, marine microorganisms residing in corals, sponges, or other marine plants also have the potential to be the sources for isolating antibiotics. Figure 7 shows several marine organisms that have become the main sources for isolating marine microorganisms, including *Streptomyces* sp., that are associated with the production of macrolides.

## 7. Concluding Remarks

TB is still a burden globally not only for its health-related impacts but also for its influence in other aspects, such as social and economic aspects. This condition is exacerbated by the ability of *M. tuberculosis* to evade the action of the current TB drugs. Efforts to discover novel drugs used to counter the resistant strains of TB should be carried out at an accelerated pace. At this point, the exploration of marine-derived compounds for their antimycobacterial activity should be taken into consideration.

The potencies of macrolides isolated from marine organisms in treating TB have attracted interests in the recent times given that several antibiotics for TB, e.g., isoniazid, ethambutol, and the currently used macrolides, show increased rates of resistance against the microbe. It becomes clear that the ocean is storing an enormous number of compounds that are unique not only in terms of their structure but also their biological activities, compared to their counterparts originating from terrestrial organisms.

To date, the number of marine-derived compounds that have been proven to possess antimycobacterial activities is minimal. However, these activities are mostly investigated through various in vitro assays, whereas in vivo, in silico, and clinical studies of marine macrolides for countering TB are very limited. Obviously, this condition should direct the research on this topic to be more expanded, with further studies carried out to decipher the antimycobacterial potencies of the compounds in in vivo and clinical settings.

Studies aiming to investigate the molecular mechanism of the compounds should also be considered. It is also obvious that studies focusing on synthesizing marine natural marine products possessing activity against mycobacterial infections have to be carried out to avoid excessive marine exploitation. 

## Figures and Tables

**Table 1:** Metabolic-engineering strategies for improving the production of marine macrolides.

Compound	Strain	Strategy	Key Findings	Ref.
Erythromycin	*Saccharopolyspora erythraea*	Inactivate a number of TetR-family transcriptional regulator (TFR) candidates.	Overexpression of SACE_7301 in wild-type and industrial *S. erythraea* strains enhanced erythromycin yields.	[51]
Pikromycin	*Streptomyces venezuelae*	Manipulate three key enzymes of branched-chain amino-acid (BCAA) catabolism, branched-chain α-keto acid dehydrogenase (BCDH), acyl-CoA dehydrogenase, and 3-ketoacyl acyl carrier-protein synthase III.	Overexpression of BCAA resulted in the highest titer of total macrolide production of 43 mg/L, which was about a 2.2-fold increase compared to that of the wild type.	[52]
Nargenicin A1	*Nocardia* sp. *CS682*	Increase the pool of precursors for glycosylation.	Over-expression of the ACCase complex enhanced the titer.	[53]
Epothilone	*Sorangium cellulosum* and *Burkholderiales strain DSM 7029*	Introduce the epothilone biosynthetic gene cluster from the myxobacterium *Sorangium cellulosum* to the chromosome of *Burkholderiales strain DSM 7029* by transposition.	Overexpression of rare tRNA genes and introduction of the exogenous methylmalonyl-CoA biosynthetic pathway elevated the total yields of epothilones to 307 μg/L.	[54]
Galbonolide B	*Streptomyces* sp. *LZ35* and *S. coelicolor*	Introduce the galbonolide-expression constructs (gbnA–E) to the host strain *S. coelicolor* ZM12 by intergeneric conjugation and integrate it into the chromosome.	Galbonolides B was successfully produced by heterologous expression.	[55]
Quinolidomicin	*Micromonospora* sp. *JY16* and *Streptomyces lividans*	Introduce LAL regulator genes (qnmRI and qnmRII) from *Micromonospora* sp. JY16 into *S. lividans* TK23ΔredDX::pKU518quiP9-L5 to activate the transcription level of the biosynthetic genes.	Heterologous expression of the biosynthetic gene cluster for quinolidomicin A1 as the 213.7 kb region. The yield of quinolidomicin A1 was approximately 0.1 mg/L in the culture broth.	[56]

**Table 2:** Several marine macrolides exhibiting anti-mycobacterial activities.

Compound	Source	Key Findings	Ref.
Jasplakinolide	Marine sponge *Jaspis johnstoni*	Jasplakinolide is a stabilizer of the actin filament. It could modulate the activity of actin microfilament, leading to the optimization of iNOS transport from the cellular membrane to the phagosome, followed by a sufficient production of NO to kill the bacterium. Unlike other phagosomes ingesting *E. coli* or other neutral molecules, the mycobacterial phagosome does not colocalize with iNOS. At this point, iNOS must be recruited from the cellular membrane of the macrophage to the phagosome. This recruitment is facilitated by the action of actin microfilament. In the study conducted by Miller et al., jasplakinolide did not induce iNOS migration to the phagosome. The same case was also seen in the administration of cytochalasin D (an actin microfilament disrupter). However, when these two compounds were combined, the transport of iNOS to the phagosome increased significantly [100].	[100,101]
Lobophorins	Marine-derived *Streptomyces* sp. MS100061	Lobophorin A, B, and G showed strong activity against *M. bovis* Bacillus Calmette-Guérin (BCG), with MIC values of 1.56, 1.56, and 0.78 μg/mL, respectively. These compounds also possessed moderate activity against *M. tuberculosis* H37Rv, with MIC values of 32, 32, and 16 μg/mL, respectively.	[102]
	Mollusk-associated *Streptomyces* sp. 1053U.I.1a.3b	Five compounds identified as compound 1 – 5 were tested for their anti-mycobacterial activity. However, the results showed that only compounds 2–5 showed strong activity against *M. tuberculosis*, with MIC_90_ of 2.6, 7.8, 1.3, and 1.4 μM, respectively. Compounds 2–5 were also tested for their cytotoxicity against a human cancer cell line (CEM-TART). The LD_50_ values were 8.6, 0.3, 1.6, and 1.7 μM, respectively.	[103]
Antimycin A and its analogues	Marine-derived actinomycete *Streptomyces lusitanus*	The action of antimycin and its analogues in fighting mycobacterial infection might be related to their activity in inhibiting cytochrome *bc_1_* complex in the *M. tuberculosis*.	[104,105,106,107]
Etamycin	Marine-derived actinomycetes *Streptomyces* spp. OPMA1730	Four microbes were used to test antimycobacterial activity of the compound, i.e., *M. avium*, *M. intracellulare*, *M. smegmatis,* and *M. bovis*. The MIC values were determined based on the liquid-microdilution method. The MIC values of etamycin against those mycobacteria were 0.097, 0.19, 25, and 0.78 μg/mL, respectively. As a reference, the MIC values of rifampicin were 0.78, 0.012, 1.56, and 0.012 μg/mL, respectively.	[108,109]
Norfijimycin A	Its parent compound, fijimycin A, was first isolated from a marine-derived *Streptomyces* spp. CNS-575Norfijimycin A is a simplified analogue of fijimycin A	Its activity against *M. tuberculosis* H37Rv was assessed using a resazurin-based assay. The MIC_50_ observed was 5 μM.	[110]
Desertomycin G	Marine-derived actinomycete *Streptomyces althioticus* MSM3	Desertomycin G was tested for its anti-mycobacterial activity against three strains of *M. tuberculosis* (H37Rv, MDR-1, and MDR-2). For all strains, the compound showed the same MIC values of 16 μg/mL. Four cell lines were used to investigate the cytotoxicity of desertomycin G, i.e., human lung carcinoma (A549), colon carcinoma (DLD-1), human breast adenocarcinoma (MCF-7) cell lines, and healthy mammary fibroblasts, with IC_50_ values after 24 h exposure of > 10, > 10, 7.2, and 9.8 μM, respectively.	[111]
Niphimycins	Marine-derived *Streptomyces* sp. IMB7-145	Niphimycin C and niphimycin Iα were tested for their anti-mycobacterial activities using H37Rv and some clinical isolates of *M. tuberculosis*. Both niphimycins gave an MIC value of 4 μg/mL against H37Rv strain. For the clinical strains (e.g., FJ05349, FJ05060, and FJ05120), both niphimycins also showed similar activity, with MIC values of 4–16 μg/mL. Four cancer cell lines (K562, HepG2, MCF-7, and HeLa) were used to observe cytotoxicity of the tested niphimycins. The IC_50_ of niphimycin C against those cell lines ranged from 8.5–10.2 μM, whereas niphimycin Iα was 6.8–23.9 μM. As a reference, doxorubicin showed an IC_50_ of 1.1–3.5 μM.	[112]
Borrelidin	Mangrove-derived *Streptomyces rochei* SCSIO ZJ89Co-culture of marine-derived *Streptomyces rochei* MB037 and fungus *Rhinocladiella similis* 35	No effect was observed for borrelidin in inhibiting MshC (mycothiol ligase), which is a critical enzyme needed by *M. tuberculosis* for supporting its growth (IC_50_ >2 mM). Five borrelidins (A, F, G, H, and I) were tested for their cytotoxic effects against five tumor cell lines, with IC_50_ of 0.122.19, 1.21–14.6, 1.15–17.46, 0.12–2.05, and >50 μM, respectively. Cytotoxic effects of these borrelidins were also tested on two non-malignant cell lines with IC_50_ values of 0.98–1.44, 8.73–12.02, 14.27–22.75, 6.13–7.26, and >50 μM, respectively.	[113,114,115]
Divergolides A–D	Mangrove endophyte *Bruguiera gymnorrhiza*	Divergolides A–D showed a promising antimycobacterial activity tested against *M. vaccae*. This investigation was carried out by using the paper-disk inhibition-zone method. The inhibition zones generated were 19, 12, 11, and 12 mm, respectively. Cytotoxic effects of divergolides A–D were also investigated on 40 tumor cell lines. Of those divergolides, only divergolide D showed a promising effect, with a mean IC_50_ of 2.4 μM, whereas the other divergolides were >10 μM.	[116]
Ramariolides	Coral mushroom *Ramaria cystidiophora*	Of four ramariolides (ramariolide A–D) isolated from the coral mushroom, ramariolide A had better activity against *M. smegmatis* and *M. tuberculosis* than the other ramariolides after conducting in vitro tests. The activity of ramariolide A against *M. smegmatis* was determined through the broth-dilution method with an MIC of 8 μg/mL. This MIC was the same as that shown by isoniazid. Ramariolide A was also active against *M. tuberculosis*, with IC_50_ and MIC values of 53 μg/mL and 64–128 μg/mL, respectively.	[117]
		MIC values were determined for ramariolides A–D, with the former ramariolide showed promising activity against two tested mycobacteria (*M. smegmatis* mc^2^ 155 and *M. tuberculosis* H37Rv). MIC values of ramariolide A against the tested mycobacteria were 30 and 25 μM, respectively.	[118]

## Data Availability

Not applicable.
